# Autism spectrum disorder in ICD-11—a critical reflection of its possible impact on clinical practice and research

**DOI:** 10.1038/s41380-023-02354-y

**Published:** 2024-01-25

**Authors:** Inge Kamp-Becker

**Affiliations:** Department of Child and Adolescent Psychiatry, Psychosomatics and Psychotherapy, Philipps University Marburg, Hans-Sachs Str. 6, 36037 Marburg, Germany

**Keywords:** Autism spectrum disorders, Psychology

## Abstract

This perspective article compares and contrasts the conceptualization of Autism Spectrum Disorder (ASD) in ICD-11 and DSM-5. By guiding the user through the ICD-11 text, it is argued that, in contrast to DSM-5, ICD-11 allows a high variety in symptom combinations, which results in an operationalization of ASD that is in favor of an extreme diverse picture, yet possibly at the expense of precision, including unforeseeable effects on clinical practice, care, and research. The clinical utility is questionable as this conceptualization can hardly be differentiated from other mental disorders and autism-like traits. It moves away from an observable, behavioral, and neurodevelopmental disorder to a disorder of inner experience that can hardly be measured objectively. It contains many vague and subjective concepts that lead to non-falsifiable diagnoses. This bears a large danger of false positive diagnoses, of further increased prevalence rates, limitations of access to ASD-specific services and of increasing the non-specificity of treatments. For research, the hypothesis is that the specificity of ASD will be reduced and this will additional increase the already high heterogeneity with the effect that replication of studies will be hampered. This could limit our understanding of etiology and biological pathways of ASD and bears the risk that precision medicine, i.e., a targeted approach for individual treatment strategies based on precise diagnostic markers, is more far from becoming reality. Thus, a more precise, quantitative description and more objective measurement of symptoms are suggested that define the clinical ASD phenotype. Identification of core ASD subtypes/endophenotypes and a precise description of symptoms is the necessary next step to advance diagnostic classification systems. Therefore, employing a more finely grained, objective, clinical symptom characterization which is more relatable to neurobehavioral concepts is of central significance.

## Introduction

To date, Autism Spectrum Disorder (ASD) is usually conceptualized as a neurodevelopmental, behaviorally defined disorder whose symptoms emerge in early development, are present in multiple contexts and persist over lifespan. Although ASD emerges from a genetic and neurobiological background, defining biological markers has not been identified. The diagnostic decision still relies on direct observation, reported behavior, and qualitative symptom descriptions and is thus dependent on subjective evaluations by clinicians. Despite its apparent subjective nature, clinical expertise is currently the only guarantor of maintaining diagnostic accuracy.

### Time trends in the conceptualization of autism

Over time, the definition of autism has alternated between narrower and broader views of the disorder [1]: From the first inclusion of “infantile autism” in DSM-III as one subgroup of “Pervasive Developmental Disorders” to the term ASD as a neurodevelopmental disorder in DSM-5. Lately, concepts of neurodiversity and the social model of disability have gained prominence and challenged traditional ideas about ASD as a disorder. In this conceptualization, ASD is one form of variation within a diversity of minds and the difficulties are not understood as a deficit or disorder, but rather as a poor fit between individual’s characteristics and demands by the environment [2], so that search for a cure is being rejected. As this conceptualization of ASD is defined rather by strengths than deficits, an ASD diagnosis offers the person a positive identity and active community. A gap has emerged between considering ASD a “disorder/disability” and “diversity/identity” with different implications for conceptualization of ASD as well as treatment [3].

### Increased heterogeneity

Alongside the changes in classification systems, by now heterogeneity has already increased with regard to the expression and severity of the core and associated symptoms of ASD [4–6]. Heterogeneity is further affected by a high variability in other aspects such as developmental trajectories, sex/gender, language abilities, cognitive functioning, adaptive behaviors, and comorbidities. High heterogeneity is also found in treatment response as well as in outcomes. The hitherto existing heterogeneity is discussed to hamper the precision of clinical diagnoses and affect decisions. Furthermore, it might also affect research, e.g., decreasing of effect sizes up to 80% in cognitive, EEG, and neuroanatomical studies [7] and by now, there is a lack of replication from genetic, brain imaging, EEG, and metabolic studies (e.g., [8]) (for review see: [9]). Altogether, high heterogeneity bears the risk of hindering the explanatory power of ASD diagnoses to discover drug regimens and effective behavioral treatments [6]. There have been several attempts to deal with the heterogeneity [10] as for example creating more stringent clinical criteria, dividing ASD into subgroups (e.g., high and low functioning or according to other criteria) or studying larger samples but “to date these efforts have not been successful” ([6], p. 1).

### Main critique on the ICD-11 conceptualization of ASD

In the following it will be discussed how ICD-11 will possibly impact clinical practice and research. The aim is to open a constructive debate that will help to improve precise diagnoses. The subsequent statements will be discussed for clinical concerns:

- ICD-11 defines ASD via a great amount of possible, but not mandatory features, some of which might not be detectable via direct observation. It does not give any advice concerning the number of symptoms necessary for a diagnosis.
- The clinical utility is questionable as this conceptualization can hardly be differentiated from other mental disorders and autism-like traits.
- The ICD-11 conceptualization of ASD moves further away from an observable, behavioral, and neurodevelopmental disorder (medical model) to a disorder of inner experience in sense of “identity” (social model) that can hardly be measured objectively as it contains many vague and subjective concepts (e.g., “compensation”; symptoms that are “only apparent in retrospect”). This leads to non-falsifiable diagnoses as it annuls the significance and assessability of any observable behavioral feature for ASD.
- This bears a large danger of false positive diagnoses and that prevalence rates will further increase;
- resulting in additional limitations of access to ASD-specific services and increasing the non-specificity of treatments.

Research concerns:

- In ICD-11, the specificity of ASD will be reduced and this will increase the already high heterogeneity with the effect that replication of study results will be hampered.
- This bears the risk for limiting our understanding of etiology and biological pathways of ASD.
- It implies the risk that precision medicine, i.e., a targeted approach for individual treatment strategies requires precise diagnostic classification of distinguishable samples.

By going through the ICD-11 text, the differences between ICD-11 and DSM-5 will be carved out and the ICD-11 conceptualization of ASD will be evaluated. Although much of the criticism on ICD-11 has already been leveled to DSM-5, a comparison of both conceptualizations is a necessary first step to point out the consequences of the absence of clearly defined differential criteria for ASD. All citations of ICD-11 text are from [11]. ICD-11-citations will further be labeled in italics.

## ICD-11: Conceptualization of ASD

In DSM-5 as well as in ICD-11, ASD is assigned to the category ”neurodevelopmental disorders”, characterized by impairments in cognition, communication, behavior and/or motor skills resulting from abnormal brain development. Main features of these disorders are the onset in early childhood, associated with impairments in personal, social, educational, and occupational development and that they tend to occur together [12].

## ICD-11: *Description and diagnostic requirements for ASD*

In ICD-11 [11], ASD is characterized by *persistent deficits in the ability to initiate and sustain reciprocal social interaction and social communication and by a range of restricted, repetitive, and inflexible patterns of behavior, interests, or activities that are clearly atypical or excessive*. These deficits *are usually a pervasive feature of the individual’s functioning observable in all settings, although they may vary according to social, educational, or other context*. As *Essential (Required) Features*, *persistent deficits in initiating and sustaining social communication and reciprocal social interactions* are named. However, unlike in DSM-5, no mandatory features of social communication and interaction are defined. This lack of clearly defined necessary criteria also occurs in other mental disorders (e.g., in Anxiety or fear-related disorders) and may be a specification of the WHO, which did not wish to have the same prescriptive criteria as DSM-5. But in other mental disorders, necessary criteria are named (e.g., Mood Episode). ICD-11 merely states that *manifestations may include limitations* in *seven listed areas* with further possibilities. For the domain of social communication and reciprocal social interaction, *19 different manifestations* are described. The domain of restrictive and repetitive behaviors comprises *16 possible features* of which several show questionable empirical evidence of specificity for ASD, such as *excessive adherence to rules (e.g., when playing games)* or *persistent preoccupation with one or more special interests, parts of objects, or specific types of stimuli (including media)*. Indeed, there is evidence that individuals with ASD have difficulties in flexibility (e.g., [13]), show adherence to learned social rules (e.g., [14]) and use visual working memory for rule learning (e.g., [15]). There is also some evidence that children and adolescents with ASD are exposed to more media consumption than their typically developing peers or other clinical groups and that the exposure starts at a younger age (e.g., [16, 17], including negative effects on sleep, physical health, social competence (e.g., reduced ability to read facial cues) resulting in “autism like-traits” [16, 18–20]. However, the way in which ICD 11 phrases these aspects does not depict the current state of research about reliable symptoms of ASD.

Interestingly, features that are mostly associated with low-functioning or non-verbal ASD at younger age (such as flipping objects, preoccupation with unusual objects, excessive smelling or touching of objects, echolalia, visual fascination with lights or movement etc.) are not named in ICD-11. This, as well as many other text passages, underline that the focus is obviously on higher-functioning and older individuals, and not on younger children and those with intellectual and/or verbal impairment.

Some of the ICD-11 named features concern the differentiation between ASD and anxiety disorders. For example, the feature *Lack of adaptability to new experiences and circumstances, with associated distress*, is hardly distinguishable in childhood from the concept of behavioral inhibition which represents a strong risk factor for anxiety [21], and from anxiety itself. However, in another section (*Boundary with Normality (Threshold)*), it is stated that shyness or behavioral inhibition are not indicative of ASD. Additionally, anxiety is named as a *prominent symptom* of ASD in middle childhood, as well as *Social Anxiety Disorder, school refusal, and Specific Phobia*. These contradictions in the description might convey confusion rather than clarify diagnostic criteria and enhance differentiation.

A further *Essential (Required) Feature* of ASD, according to ICD-11, is that the onset of the disorder occurs during the developmental period. However, in five text passages, the significance and assessability of this requirement are limited by statements that first symptoms *become fully manifest until later* (2×) or *may only be apparent in retrospect* (2×), or *may not be detected until school entry or adolescence*. With the introduction of DSM-5, a time criterion for the onset of first symptoms of ASD was relinquished. Nevertheless, DSM-5 states that if there is “any report (of parents or another relative) that the individual had ordinary and sustained reciprocal friendships and good nonverbal communication skills” [12] p. 64) during childhood, this would exclude the diagnosis of ASD. ICD-11 does not include any such hints. In contrast, many passages suggest that an ASD diagnosis might be reasonable even in the absence of defined behavioral symptoms or impairment, e.g., *some individuals with Autism Spectrum Disorder are able to function adequately in many contexts through exceptional effort, such that their deficits may not be apparent to others. A diagnosis of Autism Spectrum Disorder is still appropriate in such cases*. This leads to non-falsifiable diagnoses, as it annuls the significance and assessability of any observable behavioral feature for ASD.

Altogether, *304 (19* *×* *16) combinations of manifestations* are possible. For example, if an individual has exhibited limitations in the ability to make and sustain typical peer relationships and has a “persistent preoccupation with special interest” in media, the diagnostic criteria for ASD according to ICD-11 would be fulfilled. The current taxonomic descriptions in ICD-11 allow for manifold manifestations, so that any arbitrary symptom presentation would justify an ASD diagnosis. A further increase in diagnoses and a dramatic increase in heterogeneity is hypothesized.

## ICD-11: *Specifiers for characterizing features within ASD*

ICD-11 retains a multi-categorical system to differentiate individuals with varying levels of developmental history (i.e., regression) and intellectual and language abilities by offering eight subcategories of ASD diagnoses. In contrast to DSM-5, ICD-11 has no specifier for severity that could provide additional information about the degree of support needed in identified areas of deficits. Maybe the reason for this was that the severity metric has shown questionable validity [22] or that in the conceptualization of ASD in ICD-11 severity in sense of impairments in functionality do not need to be present as implied in the previous statement that *some individuals with Autism Spectrum Disorder are able to function adequately in many contexts through exceptional effort, such that their deficits may not be apparent to others*.

## ICD-11: *Additional clinical features*

A lot of other mental disorders are named as *prominent* or *presenting* features for ASD: *anxiety, social anxiety, school refusal, specific phobia, depression*. It is stated that *a co-occurring disorder … first brings an individual with Autism Spectrum Disorder to clinical attention*. This emphasis on concomitant conditions might lead to the impression that any symptom combination might be associated with ASD. This is a mere apprehension based on the textual basis of ICD-11. However, if clinicians stuck to the ICD-11 definitions as revised here, this would lead to misinterpretations of differential diagnoses as ASD and, therefore, false positive diagnoses.

In the ICD-11 manuscript, *compensation* is mentioned several times (e.g., *Clinical presentation may occur when social demands overwhelm the capacity to compensate …. Compensation strategies may be sufficient to sustain dyadic relationships*). Even though compensation is a term widely used, there is currently no agreed definition [23] and remains a speculative hypothesis in diagnostic settings. Thus, this concept has been criticized as it calls into question the validity and utility of the current behavioral diagnosis of ASD [24] and results in “quasi-autism“ [25]. The assumed underlying mechanisms, specificity, and operationalization of compensation are highly variable and altogether the empirical evidence is questionable.

## ICD-11: *Boundary with normality (threshold) and developmental presentation*

It is emphasized that symptoms of ASD *may only be recognized as indicative of Autism Spectrum Disorder in retrospect*”. This statement disregards the possibilities of retrospective recall biases or that the retrospective may be affected by inaccurate caregiver memory [26–31]. This, in particular may be the case when the ASD diagnosis is possibly experienced as less burdensome or stigmatizing than other diagnoses [32, 33]. Additionally, there is some evidence that there is low agreement between diagnoses based on anamnestic interviews (ADI-R) and those based on behavioral observations, particularly for older and atypical cases, and for better performance of the behavioral observation ([22, 30, 34–40].

Interestingly, early, predictive, and well-established features of ASD as for example deficits in joint attention behavior (e.g., [41, 42]) are not mentioned in ICD-11. Instead, every paragraph of the *Development Presentation* section emphasizes that symptoms may be *overlooked, overshadowed, or compensated*. This is particularly interesting, as this possibility applies to all mental health diagnoses, but is only stressed to this extent for ASD.

## ICD-11: *Boundaries with other disorders and conditions (differential diagnosis)*

In this section, many more differential diagnoses are named than in DSM-5, which is useful and necessary.

With regard to differential diagnosis, ICD-11 emphasizes that symptoms of ASD have to be persistent and pervasive with onset in early childhood. These statements are important and helpful in the differentiation of ASD from many other diagnoses. However, the statements in earlier sections, that symptoms of ASD may be compensated, overshadowed, be presented by features of other disorders or may only be recognized in retrospect, relativize this important condition and may lead to confusion for the readership.

## Discussion

Although the conceptualization of ASD in DSM-5 is closer to ICD-11 than DSM-4, in contrast to DSM-5, ICD-11 allows a vast variety of possible symptoms, which results in an operationalization of ASD that is in favor of an extremely diverse picture, yet possibly at the expense of precision and specificity, including unforeseeable effects on clinical practice, care, and research. With the aim to increase the sensitivity for cognitively able and older individuals [43], *specificity is reduced and will further increase the already high heterogeneity*. This carries the risk that like a vicious circle, the hope of finding valid biomarkers is additionally hampered. As ICD-11 defines ASD in a broad constellation of symptoms or behaviors that can hardly be differentiated from other mental disorders and autism-like traits, the risk of false positive ASD diagnoses increases significantly. This will lead to further limitation of access to ASD-specific services for individuals with a true positive diagnosis of ASD and likewise disadvantages individuals with a false positive diagnosis of ASD due to delays in access to or even missing out on disorder-specific care (e.g., dialectical behavior therapy for borderline personality disorder). Further, since ICD-11 draws particular attention to high-functioning (adult) individuals with ASD, there is concern that “prototypical” [44] as well as low-functioning cases increasingly become neglected in research and clinical practice. This leads to the lively and vital discussion about heterogeneity and prototypes presented elsewhere [5, 44–49], which is not the subject of this perspective article.

### Implications for clinical practice

One main criticism of the ICD-11 delineation of ASD is that its clinical utility is questionable. It remains to be seen how clinicians who are not familiar with ASD will receive this conceptualization that is moving further away from an observable, behavioral, and neurodevelopmental disorder (medical model) to a disorder of inner experience in sense of “diversity” and “identity” (social model). There are several controversies surrounding the social model and consequences of early intervention methods [50–54]. This is an important and necessary discussion, but it seems questionable that a classification system is the appropriate instrument for addressing this open debate, as the Advisory Group emphasized that the focus of the ICD-11 was on the classification of *disorders* and not the assessment and treatment of individuals ([55], p. 91).

ICD-11—for the first time—mentions compensational effort several times without any definition or limitations. In contrast, the DSM 5 conceptualization makes it much clearer that there have to be observable symptoms as it states that the “impairment may be relatively subtle within individual modes” (e.g., eye contact) “but noticeable in poor integration of eye contact, gestures, body posture, prosody, and facial expression” [12] p. 61). This statement is in line with studies demonstrating that—as compensational effects are associated with cognitive and executive functions [56, 57]—basic social communication skills (especially eye contact, facial expressions, gestures, and shared enjoyment) are not associated with age and intelligence in diagnostic settings [58–60]. There is no doubt that compensation is an important concept and many individuals learn to compensate their intuitive deficits with the help of targeted interventions. However, the emphasis of compensation in ICD-11 for attention within a diagnostic procedure poses the risk that the value of standardized behavioral observations and interviews with caregivers might decrease and the criterion of an early onset of ASD might be undermined.

One reason—among others—for the revision of ICD-10 was the artificial and inflated comorbidity between mental disorders [61]. In ASD, the amount of comorbidity has increased over time, especially in late-diagnosed females [62, 63]. Around 80% of individuals with ASD have at least one comorbid disorder [64]. Often, these diagnoses are given simultaneously and “may be explained as “false positive” ASD diagnoses where conditions such as mood disorders, psychosis or eating disorders result in symptoms that are easily mistaken for ASD or temporarily amplify existing sub‐clinical autism‐like traits.” ([24] p. 483). An essential and appropriate differentiation between ASD and autism-like traits [65]—which are present in many mental or behavioral disorders (e.g., in ADHD [66] or social anxiety disorders [67] as well as in healthy individuals [68]—could be threatened with the conceptualization of ASD in ICD-11. Polemically speaking, ICD-11 opens the door for clinical practice to diagnose many mental or behavioral disorders as ASD, as “everyone is on the spectrum somewhere” [69] (p. 13). In other words: By considering “social traits” a priori as “autistic (like) traits”, any differentiation of mental disorders that are associated with social interaction problems becomes a lost cause. On the other hand, the non-specificity of the ICD-11 ASD criteria is found at the two extremes of intelligence and adaptive behavior skills, meaning that the inability to differentiate ASD also affects diagnoses of severe neurodevelopmental disorders (e.g., profound disorders of intellectual development).

One could argue, that the description of ASD in ICD-11 includes the ASD phenotype in all its facets and this may increase sensitivity, and therefore, many more individuals will get access to health care. On the other hand, without clearly defined criteria in each domain of ASD symptomatology, the differentiation to other mental and behavioral disorders will become extremely difficult for clinicians as it results in too much overlap with other disorders and hampers the differentiation to autism-like traits in the course of other disorders.

Another argument might be that all disorders include heterogeneous symptoms, gene variants, significant comorbidity, great overlap among each other and large variability. Therefore, the diagnostic boundaries of ASD in ICD-11 are rather broad due to the large variability in constellations of symptoms and developmental courses as well as heterogeneity of the biological underpinnings. Future research is needed to clarify whether an increase of heterogeneity as implied in ICD-11 brings progress in clinical contexts or solely confuses diagnostic assignment resulting in increased prevalence rates and lowered access to support systems. Although, there is no doubt that diagnosing ASD (or ruling it out) is a comprehensive and complex process which should never be solely based on counting ICD or DSM criteria, the use of standardized instruments can lead to the feeling of “false security” [70] and, thus, should be considered very thoroughly in advance.

### Implications for research

Clinical and biological research is usually done utilizing case-control comparison methodology. Groups built on ICD-11 criteria will consist of a highly heterogeneous mixture of very different types of individuals, so that replication of studies is further hampered. As the differentiation to many other mental and behavioral disorders will become extremely difficult, ICD-11 will also hamper much needed studies into comorbidities, when study samples might mix comorbidities with actual differential diagnosis. One could argue, that by presenting a very broad range of symptoms or symptom constellations it is possible to create a “feature-rich” sample and on this basis one could increase the likelihood to explain clinically or mechanistically important phenotypes with big data approaches that are “broad” (i.e., large sample size) and “deep” (i.e., multiple levels of data collected on the same individuals) [4]. But up to now, autism research is already facing a great complexity of the disorder, with indefinite results of genetic, neurobiology, mechanisms/pathophysiology and other aspects, issues of replication sample size and representativeness. Alas, the question arises whether a classification system is the appropriate instrument for addressing the problem of heterogeneity as the “usefulness of the classification as an organizing framework for research should not be confused with the scientific basis of the classification itself” ([55], p. 89).

In contrast, it is further hypothesized that increased heterogeneity bears the risk for limiting our understanding of ethology and biological pathways of ASD, resulting in a lack of diagnostic precision (i.e., reliability) and reducing the fit of therapeutic interventions. High heterogeneity holds the risk that this results in high individual variation in response to a given treatment and considerable non-specificity of treatments. This applies to pharmacological as well as behavioral treatments. It also implies the risk that precision medicine, i.e., a targeted approach for individual treatment strategies based on precise diagnostic markers, is more far from becoming reality. In my view, a more precise, multi-dimensional symptom description of the ASD phenotype, grounded in clinical utility but tightly linked to neurobehavioral measures, is necessary to identify subtypes, improve diagnostics, and to be able to apply tailored interventions.

## Outlook

Efforts to resolve the problem of insufficient reliability and validity of traditional classification systems or taxonomies have led to two opposing strategies: A) Moving from categories to dimensional descriptions of psychopathology using clinical signs and symptoms as basic blocks, and summarizing these into overlapping problem areas, syndromes and “spectra” of psychopathological syndromes (see e.g., HiTOP, [71]). The aim of such approaches is to move towards a quantitative nosology of psychopathology. B) Reconceptualising mental disorders as brain disorders, thus focusing research on specific neurobehavioral “domains” with an established link between behavioral measures and associated neural systems, and investigate their disturbance by the genetic, molecular, and neural circuit levels and how such disturbance translates into behavior and self-report measures, and, ultimately into diagnostic categories (e.g., the RDoc approach [72]). The aim of such approaches is to replace subjective nosology with specific, causal biological pathways. I argue that a refined combination of both approaches will be necessary to advance clinical care and allow for moving towards an era of precision medicine. To this day, there is a large gap between basic neuroscience research focused on neurobehavioral systems (assessed by specific laboratory-based paradigms) and clinical research focused on symptoms, maladapted behavior, and treatment (assessed by clinical evaluation and dependent on subjective evaluations). In order to bridge this gap, a joint and shared unit of observation is necessary, grounded in clinical and biological reality. Clinical rating measures need to be complemented by and linked to precise quantitative and/or qualitative measurement of symptoms on the one hand, and cognitive neuroscience measures/paradigms need to be adapted to reflect the clinical reality of atypical behavior on the other hand. For example, for precise phenotyping of ASD could be undertaken by automated identification of recognizable behavioral features in fine-grained, clinically relevant, social-interactive tasks and every-day assessments. Altogether, what we need is a more precise phenotyping in ASD and there are – to my concern - many doubts that ICD-11 is helpful in this direction. Instead, we need clearly defined, more objective criteria to 1) pinpoint the core ASD symptomatology, 2) to provide rules for assigning overlapping symptom signs to either ASD, a co-occurring condition, or both.

Identification of core ASD subtypes/endophenotypes and a precise description how these are shaped by both biological pathways and additional clinical features, is the necessary next step to advance diagnostic classification systems. Moving beyond an increasingly diffuse unitary phenotypic “Spectrum” concept towards precise clinical symptom phenotyping is essential to link symptom domains to neurobehavioral mechanisms, biological pathways and etiologies of ASD.
